# *Clostridioides difficile* associated peritonitis in peritoneal dialysis patients – a case series based review of an under-recognized entity with therapeutic challenges

**DOI:** 10.1186/s12882-020-01734-8

**Published:** 2020-03-04

**Authors:** Kairav J. Shah, Kartikeya Cherabuddi, Kalynn B. Pressly, Kaitlyn L. Wright, Ashutosh Shukla

**Affiliations:** 1 Metro Infectious Disease Consultants, 7444 Hannover Pkwy Ste 210, Stockbridge, GA 30281 USA; 2 Department of Medicine, Division of Infectious Diseases and Global Medicine, University of Florida, Gainesville, FL USA; 3 University of Florida – College of Medicine, Gainesville, FL USA; 4 Department of Veteran Affairs, North Florida South Georgia VHS, Gainesville, FL USA; 5 Department of Medicine, Division of Nephrology, Hypertension & Renal Transplantation, University of Florida, Gainesville, FL USA

**Keywords:** Peritonitis, *Clostridioides difficile*, Peritoneal dialysis

## Abstract

**Background:**

Initial presentation of peritoneal dialysis associated infectious peritonitis can be clinically indistinguishable from *Clostridioides difficile* infection (CDI) and both may demonstrate a cloudy dialysate. Empiric treatment of the former entails use of 3rd-generation cephalosporins, which could worsen CDI. We present a logical management approach of this clinical scenario providing examples of two cases with CDI associated peritonitis of varying severity where the initial picture was concerning for peritonitis and treatment for CDI resulted in successful cure.

**Case presentation:**

A 73-year-old male with ESRD managed with PD presented with fever, abdominal pain, leukocytosis and significant diarrhea. Cell count of the peritoneal dialysis effluent revealed 1050 WBCs/mm^3^ with 71% neutrophils. *C. difficile* PCR on the stool was positive. Patient was started on intra-peritoneal (IP) cefepime and vancomycin for treatment of the peritonitis and intravenous (IV) metronidazole and oral vancomycin for treatment of the *C. difficile* colitis but worsened. PD fluid culture showed no growth. He responded well to IV tigecycline, oral vancomycin and vancomycin enemas. Similarly, a 55-year-old male with ESRD with PD developed acute diarrhea and on the third day noted a cloudy effluent from his dialysis catheter. PD fluid analysis showed 1450 WBCs/mm^3^ with 49% neutrophils. IP cefepime and vancomycin were initiated. CT of the abdomen showed rectosigmoid colitis. *C. difficile* PCR on the stool was positive. IP cefepime and vancomycin were promptly discontinued. Treatment with oral vancomycin 125 mg every six hours and IV Tigecycline was initiated. PD fluid culture produced no growth. PD catheter was retained.

**Conclusions:**

In patients presenting with diarrhea with risk factors for CDI, traditional empiric treatment of PD peritonitis may need to be reexamined as they could have detrimental effects on CDI course and patient outcomes.

## Background

Peritoneal dialysis (PD) peritonitis is a dominant cause of PD failure among patients with end stage renal disease (ESRD). These individuals present largely with a gastrointestinal (GI) syndrome with or without accompanying systemic features e.g. fever; and a cloudy PD effluent secondary to elevated white blood cell (WBC) counts with predominance of neutrophils. Current management guidelines recommend early institution of empirical broad-spectrum antibiotic therapy triggered by PD fluid cytology [1]. However, even with the current recommended protocols for microbiology, 15–20% of the PD peritonitis are culture negative [2]. Concerns for atypical infections or non-infectious peritonitis are higher in culture negative peritonitis. Neutrophilic reaction can also occur with peri-peritoneal infection or inflammation as in pancreatitis and colitis [3, 4].

*Clostridioides difficile* (formerly *Clostridium difficile*) infection (CDI) presents with GI symptoms and has significant adverse outcomes including mortality, for both hospitalized and ambulatory patient populations [5–7]. Chronically ill and immunocompromised individuals, including those with end stage renal disease (ESRD), are at higher risk for developing CDI [8, 9]. These statistics along with the similar presenting syndromes, pose two unique challenges in the routine clinical care of ESRD patients on PD: GI presentation with cloudy PD effluent can detract from the timely diagnosis of CDI and empirical management of PD peritonitis with routinely recommended antimicrobials including cephalosporins negatively impacts CDI. Together, these could lead to inappropriate early management, prolonged disease course, and adverse patient outcomes [10]. A strategy that allows greater vigilance for diagnosing CDI, and appropriate antimicrobial administration without potential for worsening CDI may improve patient care in this population [11]. We present a potentially novel strategy for the empiric management of PD peritonitis through discussion of two such cases who received care in our medical center within a 12-month period.

## Case presentation

### Case #1

A 73-year-old male with Type II diabetes, ESRD managed with PD, and urethral stenosis managed by suprapubic catheter, presented to the Emergency Department (ED) with fever, abdominal pain, leukocytosis and significant diarrhea. Computed tomography (CT) of the abdomen demonstrated pan-colitis. Cell count of the peritoneal dialysis effluent revealed 1050 WBCs/mm^3^ with 71% neutrophils, 1% lymphocytes, and 28% monocytes. *Clostridioides difficile* PCR on the stool was positive. Patient was started on intra-peritoneal cefepime and vancomycin for treatment of the peritonitis and intravenous (IV) metronidazole and oral vancomycin for treatment of the *C. difficile* colitis. On day 3, due to development of ileus and worsening clinical status, oral vancomycin dose was increased to 500 mg every 6 h, vancomycin enemas were initiated along with IV tigecycline, and intra-peritoneal cefepime and vancomycin were discontinued. Peritoneal dialysate effluent culture produced no growth. IV metronidazole and vancomycin enemas were discontinued once the ileus resolved. Serial monitoring of the PD fluid with cell count was performed through day 11 and showed continued improvement in the WBC count. [Table 1] PD catheter was retained. Patient was discharged from the hospital on oral vancomycin taper after receiving 14 days of IV tigecycline.

**Table 1:** Serial PD fluid analysis and cell count

**Case #1**				
**Day**	**WBC/mm**^**3**^	**%Poly**	**%Lymph**	**% Mono**
Admission (Day Zero)	1050	71	1	28
Day 1	545	91	4	5
Day 2	93	82	8	10
Day 3	96	74	4	22
Day 4	69	88	4	8
Day 5	178	79	6	15
Day 6	237	87	1	13
Day 7	6	89	0	10
Day 8	167	70	0	30
Day 9	82	51	2	47
Day 10	40	69	5	26
Day 11	12	83	2	15
**Case #2**				
**Day**	**WBC/mm**^**3**^	**%Poly**	**%Lymph**	**% Mono**
Admission (Day Zero)	1450	49	3	48
Day 1	111	28	14	58
Day 2	58	12	10	78
Day 3	25	3	6	91
Day 4	31	4	13	83

### Case #2

A 55-year-old male with ESRD secondary to polycystic kidney disease managed with PD developed acute diarrhea ranging from ten to twenty watery bowel movements per day. He became febrile on the second day of symptoms, and on the third day he noted a cloudy effluent from his dialysis catheter and presented to the ED. He denied pain at the site of the PD catheter, and no drainage was noted at the catheter exit site. Analysis of the peritoneal dialysate effluent found 1450 WBCs/mm^3^with 49% neutrophils, 49% monocytes, and 2% lymphocytes. Intra-peritoneal cefepime and vancomycin were initiated. CT of the abdomen identified “inflammatory changes of the rectosigmoid colon compatible with an infectious or inflammatory process.” *C. difficile* PCR of the stool was positive. Intra-peritoneal cefepime and vancomycin were promptly discontinued. Treatment with oral vancomycin 125 mg every 6 h and IV Tigecycline was initiated. Serial monitoring of the PD fluid with cell count was continued. By day 3, the WBC count in effluent fluid decreased to 25 cells/mm^3^. [Table 1] PD fluid culture produced no growth. PD catheter was retained. Tigecycline was discontinued after 5 days, and the patient was discharged on oral vancomycin to complete a 14-day course. At his 2-month follow-up clinic appointment, he had fully recovered.

## Discussion and conclusions

CDI is a growing concern worldwide in both hospitalized and ambulatory patient populations. In the United States, one retrospective analysis found the incidence of CDI among hospitalized adults nearly doubled between 2001 and 2010 [12]. Further, there is worry that community-acquired CDI may be overlooked by the predominance of hospital-based studies. A population-based study in Minnesota found that community-acquired CDI accounted for 41% of all reported cases, which led the group to conclude the reported burden of disease is likely underestimated [13]. Additionally, CDI has a proclivity towards affecting those with chronic illness and immune-compromised state. As such ESRD patients with PD form a high-risk group for CDI [14].

CDI presents several unique diagnostic and therapeutic challenges in ESRD patients on PD. Although diarrhea remains the commonest symptom for CDI, overall clinical presentation of CDI can often be indistinguishable from PD peritonitis as many of these patients also present with sepsis, abdominal pain, tenderness, nausea, vomiting and diarrhea. Laroche et al. described the first case of *C. difficile* peritonitis in a patient undergoing chronic ambulatory peritoneal dialysis (CAPD) with a fatal outcome. PD fluid culture inoculated in blood culture bottles yielded *Candida albicans* and *C. difficile*. Interestingly, 3 weeks prior, the patient had received treatment for *Bacteroides fragilis* and *Streptococcus spp* peritonitis. Autopsy revealed no perforation or pseudomembranous colitis [15]. In another instance, Bharti et al. reported successful resolution of polymicrobial peritonitis in a 72-year-old man on CAPD. In this case, the PD fluid cultures using blood culture bottles revealed *E. coli, B. fragilis, C. albicans* and *C. difficile*. The identification of organisms was performed using matrix-assisted laser desorption ionization–time of flight mass spectrometry (MALDI-TOF MS) and treatment included antibiotics and PD catheter removal. As these cases demonstrate, isolation of *C. difficile* in PD peritonitis is uncommon and these are typically found in polymicrobial infections [16]. In contrast, Arikan et al. reported a 63-year-old male on PD who had previously been treated for pneumonia with piperacillin-tazobactam [17]. He presented with nausea, vomiting, abdominal pain, and cloudy dialysate and was found to have CDI, with positive stool toxin B assay, and peritonitis, with effluent white blood cell count 1160/mm^3^ with neutrophil predominance and negative fluid culture. Similarly, Ribes-Cruz et al., reported a patient with watery diarrhea, abdominal pain, fever, and cloudy peritoneal effluent that failed to respond to IP ceftazidime and vancomycin [18]. Stool was positive for *C. difficile* toxin. Peritoneal dialysate was negative for *C. difficile* toxin or antigen however, clinical improvement was noted only after the initiation of oral vancomycin. Some studies have hypothesized the concerns for transmigration of bacteria as well as bacteremia and secondary peritoneal seeding as the pathophysiology of infectious peritonitis, whereas others have suggested the upregulation of ICAM-1 receptors causing chemoattraction and transmigration of leukocytes through the intestinal epithelium causing non-infectious peritonitis [19, 20]. Fulminant CDI with perforation and peritonitis has also been demonstrated [21, 22].

**Fig. 1:**
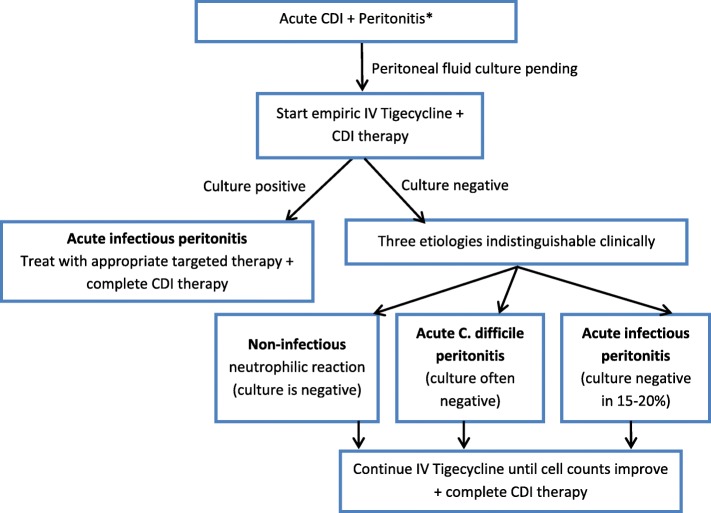
*CDI [acute diarrhea with clinical and risk factors concerning for CDI + positive stool test for *C. difficile*]; Peritonitis [clinical features including cloudy effluent + peritoneal fluid analysis with neutrophilic leukocytosis]

Taken together, these reports highlight a clinical conundrum when a PD patient presents with cloudy effluent, abdominal pain, and diarrhea likely secondary to CDI (Fig. 1). On one hand these patients may have a non-infectious neutrophilic reaction due to the presence of peri-peritoneal inflammation/colitis while on the other, the possibilities exist for a true bacterial peritonitis either with *C. difficile* itself or related to the other more conventional bacteria [4, 17, 18, 23]. Findings of cloudy effluent in these situations do not assist in establishing the diagnosis of either, and the current guidelines trigger empiric initiation of broad-spectrum antimicrobials, including third generation cephalosporins for the management of PD peritonitis. Unfortunately, these may worsen CDI. A strategy that allows for appropriate antimicrobial administration without potential for worsening CDI may improve patient care in this population [11].

Tigecycline, an intravenously administered glycylcycline, may provide an effective alternative to cephalosporins in this clinical scenario. It has antimicrobial activity covering gram-positive organisms including MRSA and VRE, gram-negative organisms as well as anaerobic bacteria and is an approved treatment of complex intra-abdominal infections including peritonitis [24, 25]. While not standard therapy for CDI, tigecycline has also been shown to have efficacy against *C. difficile* and has been reported as an effective adjunct for CDI in recent reports [26]. Herpers et al., in a case series demonstrated successful eradication of *C. difficile* in four severe refractory cases after the addition of IV tigecycline [27]. Duration of tigecycline therapy ranged between 7 to 24 days. Another retrospective cohort evaluating severe complicated, non-operative CDI showed similar outcomes in patients who received tigecycline compared to those who did not. Although, the study had a small sample size and was not powered to adjust for comorbidities and severity of illness [28]. Common side effects include nausea and vomiting while rare adverse effects of tigecycline are pancreatitis [29], hepatic dysfunction, hypersensitivity reactions with potential for low cross-reactivity to tetracyclines, photosensitivity, and pseudotumor cerebri. A black box warning exists for its use. A meta-analysis of Phase 3 and 4 clinical trials demonstrated increased all-cause mortality in tigecycline versus comparator treated patients, but the cause for this has not been determined, and use has been recommended to be reserved for specific indications [30].

In our report, Case 1 had an admission for pneumonia requiring antibiotics 1 month prior to presentation and Case 2 had a history of CDI. We used IV tigecycline as empiric treatment of possible PD associated infectious peritonitis while also providing adjunctive CDI treatment. We do not believe that tigecycline alone led to a positive treatment response but, our use of tigecycline was primarily to prevent worsening of *C. difficile* colitis. This enabled the treating physicians to avoid systemic administration of third generation cephalosporins while culture results on the dialysate fluid were awaited. The difference in duration of tigecycline between the two cases (14 days v/s 5 days) was because Case 1 had severe *C. difficile* disease and the peritoneal fluid cell count responded at a much slower rate as displayed in Table 1. Both our cases were eventually PD culture negative and thus were successfully managed with IV tigecycline and oral vancomycin without evidence for slowly responsive or refractory peritonitis. While we do not recommend combination therapy in every such patient, we feel that the empiric use of tigecycline could be considered in patients presenting with the diagnosis of CDI, or at high risk for CDI until the cause for their cloudy dialysate can be determined. This may allow appropriate coverage for the possible infectious agents without adversely impacting the CDI in those with neutrophilic reaction. In the latter cases, once PD fluid analysis shows improvement, tigecycline may be discontinued (Fig. 1).

In conclusion, the diagnosis of CDI is challenging and could be delayed or missed in PD patients presenting with diarrhea and cloudy peritoneal effluent with positive fluid cytology suggestive of PD peritonitis. CDI associated peritonitis may be inflammatory and not necessarily infectious. In patients presenting with diarrhea with risk factors for CDI, traditional empiric treatment of PD peritonitis may need to be reexamined as it could have detrimental effects on CDI course and patient outcomes. Prospective studies are needed to evaluate the ideal treatment strategy.

## Data Availability

All available data has been shared in the manuscript.

## Abbreviations

CDI: *Clostridioides difficile* infection
CT: Computed tomography
ED: Emergency department
ESRD: End stage renal disease
GI: Gastrointestinal
IV: Intravenous
PD: Peritoneal dialysis
WBC: White blood cell
